# Supplementary material for: Food Safety and Waste Management in TV Cooking Shows: A Comparative Study of Turkey and the UK
Source: Foods. 2025 Jul 24;14(15):2591. doi: 10.3390/foods14152591 (PMC12346792; doi:10.3390/foods14152591)
Supplement: Supplementary file 1 [file foods-14-02591-s001.zip › foods-3637498-supplementary.pdf]

Supplementary Material

Table S1. Number of observations and total time interval for preparation and cooking per observation regarding food safety violations that were not listed in video content analysis.

	**UK**		**Turkey**	
**Food safety violations**	**Time interval/ observation**	**Number of observations**	**Time Interval / observation**	**Number of observations**
Using metal on a Teflon pan	13:15	32	02:43:02	7
Working in a messy or unclean environment	18:26	23	57:04	21
Cleaning the plate with a kitchen cloth	02:21:19	3		
Working with a bandaged hand	00:17:40	24		
Using a kitchen cloth for sweat	01:10:40	6		
Plating food using bare hands (without gloves and utensils)	04:31	94	41:20	29
Cleaning hands on an apron			09:59:15	2
Eating food by hand			00:46:06	26
**Total number of observations**		**182**		**85**

Table S2. Number of observations and total time interval for preparation and cooking per observation in the video content analysis of food waste behaviours (i.e., actions related to meat and vegetable consumption, categorised as edible food waste).

		**UK**		**Turkey**	
**Category**	**Food waste violations**	**Time Interval / Observation**	**Number of observations**	**Time Interval/ Observation**	**Number of observations**
Edible Food Waste	Wasting food because of their lack of knowledge about processing techniques,	20:11	21	01:10:30	17
	Using only the loin, sirloin and the other prime, choice or selective cuts of the red meat, seafood or poultry	09:25	45	04:59:37	4
	Using only some parts of the vegetables or fruits.	12:07	35	44:23	27
	Total	***04:12***	***101***	***24:58***	***48***

Inedible Kitchen Waste	Choosing packaged food that would lead to waste,	13:15	32	18:44	64
	Using wooden skewers, aluminium foil, baking paper, stretch film, etc,	03:00	141	04:31	265
	Using excessive amounts of water or liquid,		0		27
	Total	***02:27***	***173***	***03:22***	***356***
Avoidable food waste	Wasting the unused parts of the food.	04:40	91	07:35	158
	Total	**91**		**158**	
	**Total**	**365**		**562**	

Note: The total preparation and cooking times used in the calculations are 423 minutes and 57 seconds for MasterChef UK and 1,198 minutes and 29 seconds for MasterChef Turkey. All time intervals per observation are displayed in hours, minutes, and seconds.

Table S3. Number of observations and total time interval for preparation and cooking per observation in the video content analysis of food safety behaviours.

**Category**	**Food safety behaviours and violations**	**UK**		**Turkey**	
		**Time Interval / Observation**	**Number of observations**	**Time Interval / Observation**	**Number of observations**
Personal Hygiene	Wearing a uniform or any similar protective equipment,	**02:21:19**	**3**	**19:58:29**	**1**
	Wearing jewellery and/or a watch,	**03:17**	**129**	**13:19**	**90**
	Having long and/or polished nails,	**10:06**	**42**		**0**
	Total	***02:26***	***174***	***13:10***	***91***
Cross - contamination	Washing their hands after touching raw meat or fish,	**03:53**	**109**	**47:56**	**25**
	Using a different chopping board for raw meat or fish,	**04:20**	**98**	**03:33**	**337**
	Changing knives or washing them after contact with raw meat/fish,	**16:18**	**26**	**03:19:45**	**6**
	Changing spoons or washing properly after tasting the cold meal,	**10:52**	**39**	**01:19:54**	**15**
	Total	***01:34***	***272***	***03:08***	***383***
	**Total Negative observations**	**00:57**	**446**	**02:32**	**474**
Personal Hygiene	Washing their hands after touching raw meat or fish,	**03:31:59**	**2**	**06:39:30**	**3**

Correct cooking	Assessing whether meat or fish was cooked properly (by using a thermometer)	**01:10:40**	**6**		**0**
	Giving instructions about cooking time,	**07:03:57**	**1**		**0**
	Giving instructions about cooking temperature	**07:03:57**	**1**		**0**
Storage condition	Mentioning cleaning procedures.		**0**	**35:15**	**34**
	**Total Positive Observations**	**42:24**	**10**	**32:23**	**37**

Note: The total preparation and cooking times used in the calculations are 423 minutes and 57 seconds for MasterChef UK and 1,198 minutes and 29 seconds for MasterChef Turkey. All time intervals per observation are displayed in hours, minutes, and seconds
